# Most of patients with localized prostate cancer will be treated in the future? | *Opinion: Yes*

**DOI:** 10.1590/S1677-5538.IBJU.2017.04.02

**Published:** 2017

**Authors:** Paolo Dell’Oglio, Rafael Sanchez-Salas

**Affiliations:** 1 Department of Urology, Institut Mutualiste Montsouris, Paris, France;; 2 Department of Urology and Division of Experimental Oncology, URI, Urological Research Institute, IRCCS San Raffaele Scientific Institute, Milan, Italy

**Keywords:** Prostatic Neoplasms, Patients, Epidemiology

## INTRODUCTION

Localized prostate cancer (LPCa) is an heterogeneous disease extending from individuals who harbor indolent cancer, that are highly unlikely to develop metastases, to individuals with more aggressive disease, that have higher risk of metastatic burden. This would translates into different oncologic outcomes and have implications for disease management. Once the diagnosis of LPCa is established, remains challenging to identify those patients who may benefit from delayed or immediate treatment. Several options exist, from active surveillance (AS) to the whole-gland treatments (1). However, the optimal one is still unclear. To date, the percentage distribution of treatment for LPCa is around 8.4% for observation, 13.1% for ablative therapies, 28.1% for external beam radiotherapy (RT), 1.6% for brachytherapy, 45% for radical prostatectomy (RP) and 3.7% for primary androgen deprivation therapy (2).

Based on the recently findings of the PROTECT trial (3), we believe that the majority of patients with LPCa will be treated in the future. Below we analyzed several points to convince the reader of this statement.

With the introduction of PSA screening in the early 1990s, a sharp increase of earlier stage PCa at diagnosis was observed. However, the release of the United States Preventive Services Task Force statements against PSA-based screening combined with the discordant findings provided by ERSPC (4) and PLCO screening trials (5), might be the triggers of the decreased PSA testing rates observed in the medical community (6). This decrease in PCa screening might lead to a reverse stage migration towards more aggressive disease in the setting of LPCa with a consequently lower probability of deferred treatment.

To date is widely accepted that PSA has a suboptimal performance as a biomarker due to its low specificity. For this reason, several biomarkers were developed and will be developed to better identify LPCa patients at increasing risk of harboring clinically significant PCa at biopsy, who might benefit from early treatment. For example, the Prostate Health Index text (PHI), combining total, free and [-2]proPSA, was demonstrated to outperform its individual components in predicting clinically significant PCa, also in biopsy naïve setting (7), reducing unnecessary biopsy. A panel of four kallikrein markers (total PSA, free PSA, intact PSA and human kallikrein 2) was also observed to improve the prediction of high-grade PCa relative to PSA testing and to reduce the number of unnecessary biopsy (43%) at the cost of missing few high-grade cancers (8). RNA biomarkers, such as PCA3 and TMPRSS2-ERG alone or combined, have shown to improve the performance of standard clinical criteria to predict high-grade PCa on biopsy (1, 9). Finally, tissue-based prognostic biomarkers, such as Prolaris and OncotypeDX, are commercially available and were observed to be strictly related in predicting adverse pathologic and oncologic outcomes (1). In conclusion, future evidence-based demonstrating superior performance of these novel promising biomarkers compared with existing standard of care is needed to allow their progressive clinical use and, consequently, more accurate identification of LPCa with aggressive disease that need immediate treatment.

Our ability to identify clinical significant PCa has dramatically improved during the last decades with the advances in imaging techniques. Multiparametric Magnetic Resonance Imaging (mpMRI) was demonstrated to have high accuracy in detecting clinically significant PCa (range: 44-87%) (10). Moreover, its high negative predictive value (range: 63-98%) could be used to rule out significant disease, sparing unnecessary biopsy (10). However, mpMRI despite promising results in biopsy naïve patients (11), is still not considered by urological guidelines in the primary biopsy setting (1). The PROMIS (11) recently provided evidence that mpMRI was more sensitive (93%) and less specific (41%) than TRUS-biopsy (48 and 96%, respectively), for detecting clinically significant PCa in biopsy naïve setting. Moreover, using mpMRI as a triage test before biopsy, will reduce unnecessary biopsy by a quarter (11). The future results of MRI-FIRST and PRECISION trials will help to define the added value of pre-biopsy MRI in biopsy-naïve setting. It is also of note to underline how mpMRI changed the biopsy paradigm opening the doors to MRI-targeted biopsy which provides higher rate of detection of clinically significant PCa (sensitivity: 91 vs. 76%, respectively) and lower rate of detection of insignificant PCa relative to TRUS-guided biopsy (sensitivity: 44 vs. 83%, respectively) (12). All these considerations suggest that mpMRI has and will have more and more high value as part of multivariable approach to early detection of clinically significant PCa.

According to the most updated urological international guidelines (1), AS is recommended for low-risk disease and a life expectancy (LE) of more than 10 years. The aim of AS is to achieve correct timing for curative treatment minimizing the treatment-related side effects without compromising oncological outcomes. Several studies reported excellent long-term oncological outcomes for patients enrolled in AS protocols (13, 14), suggesting that AS is a valid option for selected patients with LPCa. However despite AS protocols adopt stringent inclusion criteria, the treatment-free survival rates at 15 years of follow-up range from 34 to 55% (13, 14). These findings suggest that we are still far to select the optimal candidate with certainty and calls for novel biomarkers and genetic markers. Moreover, given the increasing of elderly patients, as well as the increasing LE worldwide, it might be reasonable to postulate that the future update of these AS studies will provide a trend towards higher shift into active treatment due to higher rate of disease reclassification. In consequence, identifying predictors of reclassification (e.g. PSA value at baseline, Gleason score on confirmatory biopsy) may help the physician in the daily clinical decision-making to shift into active treatment at the right time without compromising oncological outcomes. Moreover, it is of note that the risk of unfavorable pathological characteristics at RP (misclassification) is not negligible, also in those patients with very low-risk disease (15), and is higher relative to those who undergo immediate RP. Bearing in mind the predictors of unfavorable pathological characteristics in patients eligible for AS (e.g. older age, PSA density 10 ng/mL, number of positive cores) is fundamental to select the optimal candidate to immediate vs. delayed treatment. Furthermore, when we candidate a favorable intermediate-risk PCa patient to AS, we should remember that any grade pattern 4 is associated with 3-fold higher risk of metastases compared to gleason 6 (1).

The increase of low-risk and focal PCa afterwards the introduction of PSA screening combined with the well-known side effects related to whole-gland treatments, has led to the development and spreading of more conservative approaches, namely focal therapy (FT). FT is a treatment of specific focus (targeted ablation) or limited defined region (quadrant ablation or hemiablation) (16), aiming to maintain the oncological benefit of active treatment, optimizing genito-urinary and gastrointestinal side-effects. Several studies, despite their short-term follow-up, reported excellent oncological outcomes (17) and improved postoperative preservation of sexual and urinary function relative to RP and RT with pad-free continence and potency preservation rates of 100 (IQR: 95-100) and 88.6% (IQR: 78.5-97.5) for HIFU and 100 (IQR: 100-100) and 81.5% (IQR: 69.3-88.2) for cryotherapy (17). Someone could argue that only a minority of patients, namely those with unifocal low-grade tumor, may be the real candidate to FT given the fact that around 86% of PCa patients harbor a multifocal or bilateral disease (18). However, if the multifocality is an exclusion criteria, why would we candidate patients to AS? According to the last consensus conference, also selected patients with multifocal PCa and a solitary clinically significant index lesion (16) should be considered. In this way the number of patients that could benefit from FT significantly increase. The rationale in considering also these patients stems in the natural history of PCa that seems to be linked to the index lesion that drives the spreading of metastatic PCa process in the majority of men, while low-grade lesions seem to have an indolent behavior (18). Moreover, evidence-based supported no differences in BCR between unifocal vs. multifocal in patients who underwent RP (18). According to a recent consensus conference (16), FT is an acceptable strategy up to and including Gleason 4+3. The ideal candidate for FT is a patient with good LE, with clinically LPCa and single lesion of Gleason 3+4 in a location/size favorable for FT (16). The advances in imaging and targeted biopsy allow an accurate selection of patients, that becomes mandatory to ensure the success of FT. mpMRI-TRUS fusion-guided biopsy is the modality of choice to proper select patients for FT (16) due to the high concordance between the index tumor location on biopsy and RP (18) and the reduce risk of missing clinically significant cancer relative to the TRUS-guided biopsy (12). Someone could argue that mpMRI alone is not sufficient to rule out all clinically significant PCa due to its intrinsic limitations. Again, the risk of non-detecting all clinically significant PCa is in common with prostate biopsy that allow to candidate a patient to AS. For this reason FT should not be considered a curative treatment and follow the treated patients over time is mandatory. Despite long-term oncological data are needed, given the improvement in proper patients selection, the promising results in terms of oncological outcomes, with a minimal or null impact on quality of life, as well as a shift towards extending the indication of FT for intermediate-risk PCa patients with limited targetable volume, we will expect a sharp increase of LPCa patients treated with FT.

Despite the well-known side effects and the consequently impact on quality of life, whole-gland therapies still have a dominant role in the management of LPCa patients (2) and represent the gold standard for this subset of PCa patients (1). While RT use is decreasing over time in this setting, RP remains the primary treatment of choice in contemporary patients diagnosed with LPCa (2). Moreover, the use of surgery is increasing across all risk groups with LPCa (2). One of the possible explanations is the recent spreading of the robot-assisted RP (RARP) that has largely replaced open RP as preferred approach for extirpative treatment for LPCa, due to better perioperative and functional outcomes. However, it is of note that in the most contemporary patients treated with RARP, up to 20% of patients with favorable characteristics experience urinary incontinence or erectile dysfunction (19). Moreover, the rate of postoperative complications may still reach 20% (20). In consequence, there is still need to improve and surgical expertise is one of the major determinants for this enhancement. Randomized controlled trials (RCT) have been conducted to provide insight into overall treatment strategies for LPCa. The SPCG-4 randomized study (21) compared RP vs. watchful waiting and provided evidence that overall survival, PCa survival and progression-free survival were higher in the treatment group at 18 years of follow-up. Despite the study enrolled men predominantly during the pre-PSA era, with a significant number of patients harboring palpable disease, it represents the RCT in LPCa with the longer follow-up available to date. The PIVOT trial (22) made a similar comparison relying on predominantly screen-detected LPCa patients and failed to observe a benefit in overall survival and PCa survival for treatment group, within a median follow-up of 10 years, except for patients with PSA>10 ng/ml or high-risk LPCa. However, it is reasonable to think that these findings were influenced by several factors. First, this study did not meet the pre-specified enrollment targets and in consequence is underpowered to show treatment related differences. Second, 48% of patients died during the study period, of which around 85% died from other causes. These findings suggest that probably a significant number of patients enrolled into the PIVOT trial do not satisfy the 10-year LE benchmark as proposed by urological guidelines (1). Third, considering the fact that an overwhelmingly number of indolent cancers are included relative to the SPCG-4, the follow-up is quite short to evidence differences in survival between the two groups of treatments. The findings of the first RCT assessing effectiveness of RP vs. RT vs. AS were recently published (3). Despite the AS group did not undergo to a formal AS strategy (no systematic repeated biopsy/no imaging during follow-up), the PROTECT trial (3) failed to observe differences in PCa-specific and overall mortality at 10-years of follow-up between three randomized groups. However, the rate of disease progression was less than half in RP or RT groups relative to AS one. This calls for a longer follow-up to really verify the absence of benefit of immediate treatment, especially in the presence of high rate of T1c disease and low rate of Gleason 8-10 (76 and 2%, respectively).

In conclusion, our ability to identify and stratify clinically significant PCa has dramatically improved and it is likely to improve even further. First, because of the development of new biomarkers and genetic testing. Second, because mpMRI have definitely changed PCa pathway in the last few-years. Third, and most importantly, because the combination of these tools will positively lead to an accurate selection of LPCa patients who will undergo immediate treatment. The latter combined with the improvement of FT, the availability of long-term oncological data on FT, and the increasing surgical expertise with minimally invasive approach will lead to an increase of LPCa that will be safely treated in the future with more personalized approach starting from the assumption of the inter-patient and intra-glandular heterogeneity.
